# Development and Testing of Improved Models to Predict Payment Using Centers for Medicare & Medicaid Services Claims Data

**DOI:** 10.1001/jamanetworkopen.2019.8406

**Published:** 2019-08-14

**Authors:** Harlan M. Krumholz, Frederick Warner, Andreas Coppi, Elizabeth W. Triche, Shu-Xia Li, Shiwani Mahajan, Yixin Li, Susannah M. Bernheim, Jacqueline Grady, Karen Dorsey, Nihar R. Desai, Zhenqiu Lin, Sharon-Lise T. Normand

**Affiliations:** 1 Department of Health Policy and Management, Yale School of Public Health, New Haven, Connecticut; 2 Center for Outcomes Research and Evaluation, Yale–New Haven Hospital, New Haven, Connecticut; 3 Section of Cardiovascular Medicine, Department of Internal Medicine, Yale School of Medicine, New Haven, Connecticut; 4 Section of General Internal Medicine, Department of Internal Medicine, Yale School of Medicine, New Haven, Connecticut; 5 Section of General Pediatrics, Department of Pediatrics, Yale School of Medicine, New Haven, Connecticut; 6 Department of Biostatistics, Harvard T.H. Chan School of Public Health, Harvard University, Boston, Massachusetts; 7 Department of Health Care Policy, Harvard Medical School, Boston, Massachusetts

## Abstract

**Importance:**

Predicting payments for particular conditions or populations is essential for research, benchmarking, public reporting, and calculations for population-based programs. Centers for Medicare & Medicaid Services (CMS) models often group codes into disease categories, but using single, rather than grouped, diagnostic codes and leveraging present on admission (POA) codes may enhance these models.

**Objective:**

To determine whether changes to the candidate variables in CMS models would improve risk models predicting patient total payment within 30 days of hospitalization for acute myocardial infarction (AMI), heart failure (HF), and pneumonia.

**Design, Setting, and Participants:**

This comparative effectiveness research study used data from Medicare fee-for-service hospitalizations for AMI, HF, and pneumonia at acute care hospitals from July 1, 2013, through September 30, 2015. Payments across multiple care settings, services, and supplies were included and adjusted for geographic and policy variations, corrected for inflation, and winsorized. The same data source was used but varied for the candidate variables and their selection, and the method used by CMS for public reporting that used grouped codes was compared with variations that used POA codes and single diagnostic codes. Combinations of use of POA codes, separation of index admission diagnoses from those in the previous 12 months, and use of individual *International Classification of Diseases, Ninth Revision, Clinical Modification* codes instead of grouped diagnostic categories were tested. Data analysis was performed from December 4, 2017, to June 10, 2019.

**Main Outcomes and Measures:**

The models’ goodness of fit was compared using root mean square error (RMSE) and the McFadden pseudo *R*^2^.

**Results:**

Among the 1 943 049 total hospitalizations of the study participants, 343 116 admissions were for AMI (52.5% male; 37.4% aged ≤74 years), 677 044 for HF (45.5% male; 25.9% aged ≤74 years), and 922 889 for pneumonia (46.4% male; 28.2% aged ≤74 years). The mean (SD) 30-day payment was $23 103 ($18 221) for AMI, $16 365 ($12 527) for HF, and $17 097 ($12 087) for pneumonia. Each incremental model change improved the pseudo *R*^2^ and RMSE. Incorporating all 3 changes improved the pseudo *R*^2^ of the patient-level models from 0.077 to 0.129 for AMI, from 0.042 to 0.129 for HF, and from 0.114 to 0.237 for pneumonia. Parallel improvements in RMSE were found for all 3 conditions.

**Conclusions and Relevance:**

Leveraging POA codes, separating index from previous diagnoses, and using single diagnostic codes improved payment models. Better models can potentially improve research, benchmarking, public reporting, and calculations for population-based programs.

## Key Points

**Question:**

Does leveraging present on admission codes and using single, rather than grouped, diagnostic codes enhance risk models for acute myocardial infarction, heart failure, and pneumonia payment measures?

**Findings:**

In this comparative effectiveness research study of risk models on 1 667 983 patients with 1 943 049 Medicare fee-for-service hospitalizations, use of present on admission codes and single diagnosis codes and separation of index admission codes from codes in the previous year improved models predicting payment that were compared with models based on Centers for Medicare & Medicaid Services grouped codes. The patient-level pseudo *R*^2^ improved from 0.077 to 0.129 for acute myocardial infarction, from 0.042 to 0.129 for heart failure, and from 0.114 to 0.237 for pneumonia.

**Meaning:**

Changing candidate variables from the current standard improved models predicting payments, which has implications for research, benchmarking, public reporting, and calculations for population-based programs.

## Teaser

This comparative effectiveness research study assesses whether using present on admission codes and single, rather than grouped, diagnostic codes can enhance Centers for Medicare & Medicaid (CMS) models to predict payment for hospitalization for acute myocardial infarction, heart failure, and pneumonia.

## Introduction

Predicting payments for particular conditions or populations is essential for research, benchmarking, public reporting, and calculations for population-based budgeting and payment programs. The Centers for Medicare & Medicaid Services (CMS) began publicly reporting payments for hospitalizations related to acute myocardial infarction (AMI), heart failure (HF), and pneumonia in 2014-2015. These payment measures are slated to become part of the Hospital Value-Based Purchasing in 2021. Medicare Advantage uses similar models to calculate payments.

Current CMS models predicting payments have grouped *International Classification of Diseases, Ninth Revision, Clinical Modification* (*ICD-9-CM*) diagnosis codes into clinically coherent categories,^1,2^ but using single, rather than grouped, diagnostic codes may enhance these models.^3,4^ The grouping of diagnostic codes reduces the number of candidate variables but may diminish the performance of the models by combining different codes with different relationships to the outcomes. The use of single codes rather than condition categories could provide a better characterization of risk.

Since 2014, the claims data have included present on admission (POA) codes.^5^ The POA designation enables the differentiation of conditions that are POA from those that develop during the hospitalization. Current models purposely exclude conditions that might be a prehospitalization condition or ones that occurred during the hospitalization. For example, a secondary code of bleeding before the implementation of the POA designation might have represented bleeding before admission or during the hospitalization as a complication of care. Because these codes could not differentiate many diagnoses that occurred during the hospitalization from those present at hospital admission, they were not used as candidate variables for the model. With POA codes, it is possible to know that the condition was not a complication during hospitalization, allowing the use of more diagnoses from the index with the possibility of model improvement.^6,7^

We conducted several data experiments to determine whether the use of single codes or POA designations and consideration of the timing of diagnostic codes could enhance the payment model performance. First, we tested whether using the grouped codes with the POA codes could improve the discrimination and calibration of the models. Second, we examined the changes associated with separating into different risk variables the information coded in the index admission claim from information coded in claims for health care encounters in the 12 months before the index admission. Third, we evaluated changes associated with using top-ranked single diagnostic codes in place of using grouped codes as risk variables.

## Methods

### Data Source and Study Sample

In this comparative effectiveness research study, 3 condition-specific payment cohorts (AMI, HF, and pneumonia) were constructed using Medicare fee-for-service administrative claims from July 1, 2013, through September 30, 2015, as specified by the publicly reported CMS payment measures.^8,9,10^ The Yale University Human Investigation Committee, New Haven, Connecticut, reviewed the study protocol and waived the requirement for informed consent because the research involved no more than minimal risk and could not practicably be carried out without the waiver. The study followed the International Society for Pharmacoeconomics and Outcomes Research (ISPOR) reporting guideline.

Patients younger than 65 years were excluded, but no exclusions were made based on geographic location. Total payments within 30 days of an index admission for AMI, HF, or pneumonia as an outcome were used, as described previously.^11^ Patients who died within the 30-day period were included, and their payment data are not prorated. Payments across multiple care settings, services, and supplies were included, and payments were standardized to remove geographic and policy variations^11^ and adjusted to 2015 US dollars. The standardized payment was winsorized at 99.5% to reduce the effect of outliers on model prediction. To derive model covariates, candidate risk variables were identified from the index claims and claims within the 12 months, including all inpatient and outpatient claims, before the index admission. Patients without a full 12 months of previous fee-for-service enrollment were excluded.

### Model Modifications

We focused on modifying model covariates and otherwise used methods from the publicly reported CMS payment measures.^12,13,14,15,16,17,18^ We made 3 sequential incremental changes on candidate variable construction and assessed their effect on model performance in comparison with existing models. Base models were constructed using the same modified condition categories currently used for risk adjustment in the AMI, HF, and pneumonia payment models to capture patient-level comorbidity. Modified condition category candidate variables were constructed using the 2016 version 22 hierarchical condition categories (HCCs) with modifications.^19^

The models aim to adjust for demographic factors and comorbid conditions of patients at or before admission. Because the CMS payment models were developed before hospitals consistently used POA coding, the models use a clinically vetted algorithm to exclude diagnoses present only during the index hospitalization (not in the previous 12 months) that could be complications of care during that hospitalization. The first incremental modification was to incorporate POA indicators to include conditions that were POA and exclude complications that occurred during the hospitalization. Specifically, we included index secondary diagnosis codes with a POA indicator of “yes” or “missing,” with the code on a modified POA-exempt list. The details are described in the eAppendix in the Supplement.

Building on the POA models, we constructed risk variables for all 2016 version 22 HCCs using the index admission and historical diagnoses in separate and combined variables, and these variables were combined in 3 different ways: (1) using diagnoses from the index admission only, (2) combining principal and secondary diagnoses from the index and historical admissions, and (3) creating separate version 22 variables for principal and secondary diagnoses from both the index and the historical admissions, postulating different effects based on when the diagnoses were made. In addition, the HCC groups were disaggregated, and separate history and index individual *ICD-9-CM* diagnosis codes were considered as candidate variables. For these individual code models, codes were restricted to those with more than 0.5% frequency. For all the models, age was included as a categorical variable (≤74 years, 75–84 years, and ≥85 years), as specified in the current payment models.

### Statistical Analysis

We retained the generalized linear model (GLM) approach for AMI, HF, and pneumonia currently implemented in the CMS models.^20^ For AMI, the model used an inverse Gaussian distribution and a log link. For HF, the model used the Gamma distribution with a log link, and for pneumonia, the model used the Gamma distribution with an identity link.

The least absolute shrinkage and selection operator (LASSO) method was used for variable selection for models using individual *ICD-9-CM* codes. For computational feasibility, we chose the models with 200 or fewer variables, and all final variables needed to be significant with *P* < .005 in the GLM. We used 5-fold cross-validation to control for model overfitting, performing variable selection and training a GLM on 80% of the cohort and testing on the remaining 20%. Goodness of fit of the model was compared using root mean square error (RMSE) and the McFadden pseudo *R*^2^.^21^ The RMSE is an out-of-sample prediction error and should be as close to 0 as possible. The pseudo *R*^2^ measures how much the fitted model improves on the null model with no covariates. As with the *R*^2^ encountered in least squares analysis, this quantity is bounded between 0 and 1 and increases as the model improves.

To compare calibration of different models, we calculated predictive ratios for the current CMS model and the best individual *ICD-9-CM* code–based model, defined as the ratio of the mean predicted payment to the mean actual payment for deciles of predicted payment. These ratios should be close to 1 for well-calibrated models. Additional comparisons and a list of the top *ICD-9-CM* codes selected by each model are included in eTables 1-3 in the Supplement.

We also used the CMS methods of calculating the risk-standardized payment (RSP) with a hierarchical GLM, which risk adjusts for a patient’s condition at admission and seeks to isolate payment variation that reflects practice patterns at the hospital level.^8,9^ The RSP was calculated as the ratio of predicted payment divided by expected payment times the mean national payment for each condition. We used bootstrapping with 2000 iterations to derive the 95% CI for the RSPs. Hospitals with at least 25 cases were further categorized into 3 performance categories: lower than, no different from, or higher than the national mean payment. For example, if a hospital had an RSP with a lower 95% CI limit that was greater than the national mean, it was categorized as higher than the national mean. We compared the number of hospitals in each category and assessed whether the hospital performance categories shifted using different models.

All GLM-related analyses were performed in R, version 3.4.3 (R Core Team) using the base Stats package. The LASSO analysis was implemented using the H2O library in R and lassoglm in Matlab, version 2017b (MathWorks). All hospital-level analyses were done using SAS, version 9.4 (SAS Institute Inc).

## Results

### Cohort Description

The AMI, HF, and pneumonia cohorts are described in Table 1. Among 1 943 049 total admissions, 343 116 were for AMI (52.5% male; 37.4% aged ≤74 years; and 86.8% white), 677 044 for HF (45.5% male; 25.9% aged ≤74 years; and 83.9% white), and 922 889 for pneumonia (46.4% male; 28.2% aged ≤74 years; and 87.1% white). The mean (SD) 30-day payment was $23 103 ($18 221) for AMI, $16 365 ($12 527) for HF, and $17 097 ($12 087) for pneumonia.

**Table 1.** Cohort Description for Index Admissions of Acute Myocardial Infarction, Heart Failure, and Pneumonia^a^

Characteristic	Acute Myocardial Infarction (n = 343 116)	Heart Failure (n = 677 044)	Pneumonia (n = 922 889)
Unique patients	335 046 (97.6)	610 537 (90.2)	866 179 (93.8)
Unique hospitals	4001	4378	4455
30-d Payment, mean (SD), $	23 102.6 (18 221.0)	16 364.7 (12 526.6)	17 096.9 (12 086.7)
Male	180 223 (52.5)	308 321 (45.5)	428 393 (46.4)
Age, y			
65-74	128 315 (37.4)	175 413 (25.9)	260 041 (28.2)
75-84	124 902 (36.4)	249 127 (36.8)	337 063 (36.5)
≥85	89 899 (26.2)	252 504 (37.3)	325 785 (35.3)
Race/ethnicity			
White	297 973 (86.8)	567 985 (83.9)	804 260 (87.1)
African American	27 413 (8.0)	77 435 (11.4)	69 583 (7.5)
Hispanic	5273 (1.5)	11 325 (1.7)	16 385 (1.8)
Asian	4915 (1.4)	8692 (1.3)	14 700 (1.6)
North American Native	1983 (0.6)	3484 (0.5)	6228 (0.7)
Other	4137 (1.2)	6614 (1.0)	9496 (1.0)
Unknown	1422 (0.4)	1509 (0.2)	2236 (0.2)

^a^ Data are presented as number (percentage) of admissions unless otherwise indicated.

### Performance of Patient-Level 30-Day Payment Models

Table 2 shows the pseudo *R*^2^ and the RMSE for the incremental changes to the patient-level models for AMI, HF, and pneumonia cohorts. The CMS base models had a pseudo *R*^2^ of 0.077 for AMI, 0.042 for HF, and 0.114 for pneumonia measures. Incorporating POA indicators in the CMS model had a mixed effect on the goodness of fit of the models. Models for AMI that included POA codes had worse performance compared with the current CMS publicly reported measure, producing a lower pseudo *R*^2^ (0.065 vs 0.077) and higher RMSEs (17 414 vs 17 235), but models for HF and pneumonia had better performance, with a higher pseudo *R*^2^ (0.048 vs 0.042 for HF; 0.150 vs 0.114 for pneumonia) and lower RMSEs (11 423 vs 11 449 for HF; 11 256 vs 11 382 for pneumonia).

**Table 2.** Performance Comparison of the Current Patient-Level CMS Models and Models That Incrementally Implement the Proposed Changes for AMI, HF, and Pneumonia

Data	Pseudo *R*^2^ Full Model^a^				RMSE, Mean (SD), $^a^			
	AMI	HF	Pneumonia	AMI		HF	Pneumonia
CMS							
Base model	0.077	0.042	0.114	17235.1 (89.7)		11 449.3 (57.8)	11 382.4 (35.0)
Base model incorporating POA coding	0.065	0.048	0.150	17 414.0 (86.9)		11 422.7 (56.1)	11 255.6 (36.3)
HCC							
Model using index diagnosis only	0.107	0.102	0.215	17 012.5 (83.7)		11 181.4 (57.2)	10 851.6 (30.6)
Model using pooled history and index diagnosis^b^	0.084	0.062	0.170	17 218.5 (87.5)		11 367.3 (55.7)	11 145.0 (32.4)
Model using history and index diagnosis separately	0.114	0.109	0.230	16 929.9 (79.4)		11 160.0 (56.9)	10 803.8 (29.1)
Top index admission individual *ICD-9-CM* codes selected by LASSO, frequency >.5%	0.125	0.121	0.222	16 845.6 (83.0)		11 108.2 (64.6)	10 863.3 (31.1)
Top index and history admission individual *ICD-9-CM* codes selected by LASSO, frequency >0.5%	0.129	0.129	0.237	16 804.4 (75.4)		11 080.7 (62.7)	10 797.3 (34.8)

Abbreviations: AMI, acute myocardial infarction; CMS, Centers for Medicare & Medicaid Services; HCC, hierarchical condition category; HF, heart failure; *ICD-9*-*CM*, *International Classification of Diseases, Ninth Revision, Clinical Modification*; LASSO, least absolute shrinkage and selection operator; POA, present on admission; RMSE, root mean square error.

^a^ Pseudo *R*^2^ full model calculated using full cohort and RMSE calculated using 5-fold cross-validation.

^b^ Risk factor is set to be yes if the patient had a history or index diagnosis or both.

The next 3 models included all 201 HCCs as risk variables, each incorporating the collection of historical and index diagnoses in a different manner. For all 3 measures, HCC models, including pooled history and index diagnoses (pseudo *R*^2^, 0.084 for AMI, 0.062 for HF, and 0.170 for pneumonia), performed worse than models including index diagnoses only (pseudo *R*^2^, 0.107 for AMI, 0.102 for HF, and 0.215 for pneumonia); models with separate variables for index and history HCCs performed best (pseudo *R*^2^, 0.114 for AMI, 0.109 for HF, and 0.230 for pneumonia). The RMSE values indicated similar performance changes comparing different models.

Models including individual *ICD-9-CM* codes as risk variables instead of condition categories further improved the model performance. The best performing model used both index and historical admissions, with the individual *ICD-9-CM* codes selected by LASSO among codes with frequency greater than 0.5% (pseudo *R*^2^, 0.129 for AMI, 0.129 for HF, and 0.237 for pneumonia). The RMSEs improved for the 3 measures.

Table 3 compares the predictive ratios for the CMS model and the individual-codes model. In all cases, for both models, the predictive ratios were close to 1. For AMI, the predictive ratios ranged from 0.93 (in decile 10) to 1.05 (in deciles 5 and 6) for the CMS model and from 0.96 (in deciles 8 and 9) to 1.02 (in deciles 3-6 and 10) for the individual-codes model. For HF, the CMS model predictive ratios ranged from 0.99 (in deciles 5-8) to 1.03 (in decile 1), and they ranged from 0.95 (in decile 7) to 1.08 (in deciles 1 and 10) for the individual-codes model. The range of the predictive ratios of the 2 models was similar for pneumonia, from 0.96 (in decile 5) to 1.07 (in decile 1) for the CMS model and from 0.96 (in deciles 4 and 5) to 1.08 (in deciles 1 and 10) for the individual-codes model.

**Table 3.** Ratio of Mean of Predicted Payment to Mean of Actual Payment for Deciles of Predicted Payment for Acute Myocardial Infarction, Heart Failure, and Pneumonia

Model Type	Lower to Higher Predicted Payment Deciles									
	First	Second	Third	Fourth	Fifth	Sixth	Seventh	Eighth	Ninth	Tenth
**Acute Myocardial Infarction**										
CMS	0.95	1.00	1.01	1.03	1.05	1.05	1.04	1.01	0.95	0.93
Individual-codes	0.99	1.01	1.02	1.02	1.02	1.02	1.00	0.96	0.96	1.02
**Heart Failure**										
CMS	1.03	1.01	1.00	1.00	0.99	0.99	0.99	0.99	1.00	1.02
Individual-codes	1.08	1.05	1.01	0.98	0.97	0.96	0.95	0.97	0.99	1.08
**Pneumonia**										
CMS	1.07	1.00	0.98	0.97	0.96	0.97	0.98	1.00	1.02	1.06
Individual-codes	1.08	1.00	0.97	0.96	0.96	0.97	0.98	1.00	1.03	1.08

Abbreviation: CMS, Centers for Medicare & Medicaid Services.

The Figure shows the distribution of patient-predicted payments across the 3 cohorts. The individual-codes models had a wider range of predicted payments compared with the CMS models. Specifically, the median payments predicted by the individual-codes model were $21 712 (range, $8204-$113 447) for AMI, $15 307 ($8432-$80 447) for HF, and $16 207 ($5402-$59 155) for pneumonia, whereas those medians predicted by the CMS models were $22 423 ($10 994-$55 387) for AMI, $15 805 ($12 612-$36 766) for HF, and $16 529 ($8285-$41 126) for pneumonia. More detail on how the distribution of predicted payments differed between the 2 models is given in eTables 4-6 in the Supplement, in which the models’ quintiles of predicted payment and how patients are reclassified by each of the 2 models are compared. We found that the individual-codes model predicted lower values in the bottom quintiles and higher values in the top quintiles for all 3 conditions, while maintaining a similar level of discrimination and goodness of fit. To quantify this for AMI, although the middle quintiles of the 2 distributions of predicted payment had similar outcome rates, the bottom quintile had an outcome rate 9.0% lower in the individual-codes model than in the CMS model; the rate was 5.4% higher in the top quintile for the individual-codes model than in the CMS model. For HF, the bottom quintile was lower by 16.1% and the top quintile was higher by 12.2%, and for pneumonia, the corresponding changes were 15.3% and 10.8%.

**Figure.**
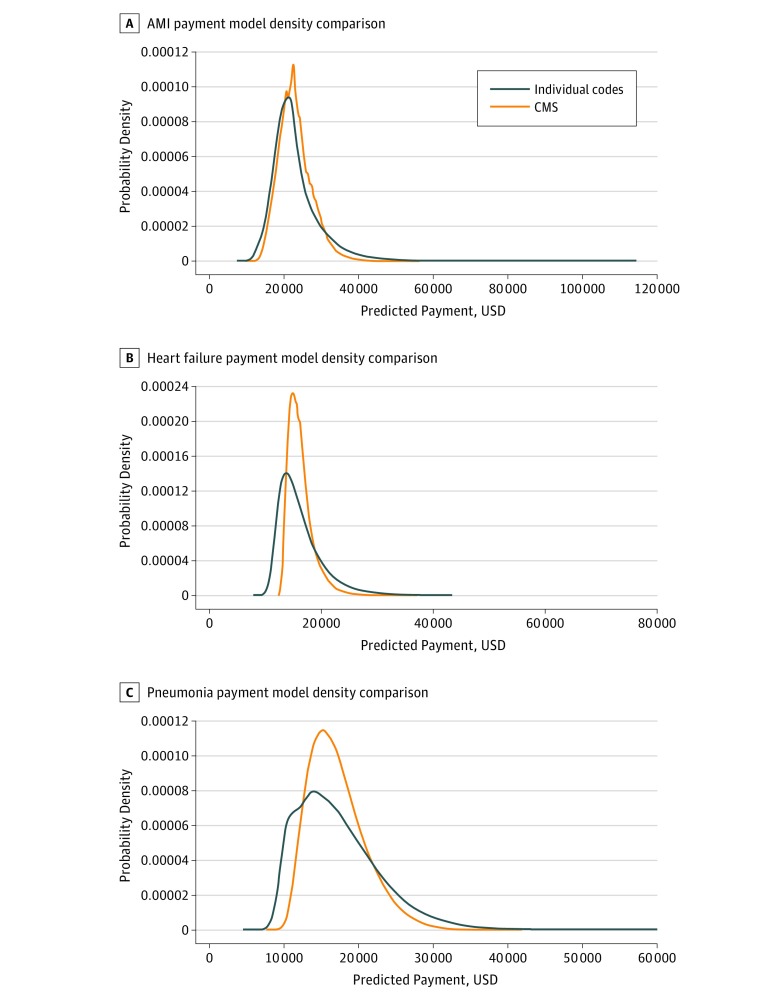
Kernel Density Plots of Predicted Payment Comparing the Centers for Medicare & Medicaid Services (CMS) Model With the Individual *International Classification of Diseases, Ninth Revision, Clinical Modification* Codes Model for Acute Myocardial Infarction (AMI), Heart Failure, and Pneumonia 30-Day Payment Measures

### Comparison of Hospital Performance

A total of 4001 hospitals contributed claims for the AMI cohort, 4378 for the HF cohort, and 4455 for the pneumonia cohort; of these, 2181 hospitals for AMI, 3265 for HF, and 3831 for pneumonia had at least 25 cases of each disease. The distribution of RSPs using individual *ICD-9-CM* codes had similar or smaller variations compared with those using the CMS model (Table 4). The mean (SD) RSP for the CMS model was $23 105 ($1589) compared with $23 211 ($1567) for the individual-codes model. The mean (SD) RSP for HF was $16 294 ($1309) in the CMS model compared with $16 230 ($1112) in the individual-codes model and for pneumonia was $17 065 ($1809) in the CMS model compared with $17 057 ($1616) in the individual-codes model. Mean (SE) differences of individual hospital RSPs, calculated as CMS RSPs minus individual *ICD-9-CM* code RSPs, were $106 ($16) for AMI, $571 ($10) for HF, and $8 ($19) for pneumonia.

**Table 4.** Hospital-Level 30-Day Payment Measures for Acute Myocardial Infarction, Heart Failure, and Pneumonia, by Model Type, Among Hospitals With at Least 25 Cases

Condition, Model Type	RSP, Mean (SD), $	RSP, Median (IQR) [Range], $
Acute myocardial infarction (n = 2181)		
CMS	23 211 (1567)	23 179 (22 150-24 228) [13 882-30 176]
Individual-codes	23 105 (1589)	23 077 (22 060-24 169) [13 476-29 648]
Heart failure (n = 3265)		
CMS	16 294 (1309)	16 197 (15 397-17 085) [11 872-21 714]
Individual-codes	16 230 (1112)	16 162 (15 448-16 907) [12 140-20 723]
Pneumonia (n = 3831)		
CMS	17 065 (1809)	17 035 (15 935-18 079) [10 295-26 116]
Individual-codes	17 057 (1616)	16 827 (16 042-17 769) [11 467-27 411]

Abbreviations: CMS, Centers for Medicare & Medicaid Services; IQR, interquartile range; RSP, risk-standardized payment.

Numbers and proportions of hospitals categorized as no different from the national mean for payment using CMS models and the individual-codes models were similar (1861 [85.3%] vs 1823 [83.6%] for AMI, 2538 [77.7%] vs 2615 [80.1%] for HF, and 2435 [63.6%] vs 2544 [66.4%] for pneumonia) (eTable 7 in the Supplement). There were fewer hospitals categorized as lower than the national mean for payment for all 3 conditions using the CMS model compared with the individual-codes model (161 [7.4%] vs 227 [10.4%] for AMI, 287 [8.8%] vs 320 [9.8%] for HF, 713 [18.6%] vs 807 [21.1%] for pneumonia). However, there were more hospitals in higher than national payment categories using the CMS model compared with the individual-codes model (159 [7.3%] vs 131 [6.0%] for AMI, 440 [13.5%] vs 330 [10.1%] for HF, and 683 [17.8%] vs 480 [12.5%] for pneumonia). Compared with the CMS model, the individual-codes model resulted in a shift of 24 (AMI), 97 (HF), and 285 (pneumonia) hospitals from lower than the national payment to no different from the national payment; a shift of 73 (AMI), 163 (HF), and 335 (pneumonia) hospitals from higher than the national payment to no different from the national payment; a shift of 90 (AMI), 130 (HF), and 365 (pneumonia) hospitals from no different from the national payment to lower than the national payment; and a shift of 45 (AMI), 53 (HF), and 146 (pneumonia) hospitals from no different from the national payment to higher than the national payment. There were no hospitals that shifted between lower than the national payment and higher than the national payment categories except the 14 hospitals for pneumonia (eTable 8 in the Supplement). Summarizing this in percentage terms, hospital reclassifications were 10.6% (232 of 2181 for AMI), 13.6% (443 of 3265 for HF), and 29.9% (1145 of 3831 for pneumonia). Also, there were 2163 hospitals with qualifying numbers of admissions for all 3 conditions, and 49.1% (1062 of 2163) were reclassified for at least 1 condition.

## Discussion

In this study, payment models for Medicare beneficiaries using individual codes instead of condition categories and leveraging the new POA designations outperformed models based on grouped diagnoses and not leveraging POA codes. The individual-codes models produced better discrimination, goodness of fit, and predictive range, with similar calibration. The improvement was most evident in identifying patients at the upper and lower ends of cost. Moreover, the new models achieved these improvements without any additional data collection and only involved a change in the incorporation of covariates into the models.

For decades, researchers and policymakers used diagnostic groups to organize different diagnostic codes into a small set of categories based on clinical sensibility. The rationale was to summarize data based on patterns of care.^22^ In 1989, Ash and colleagues^23^ introduced a diagnostic cost group approach to a reimbursement model for health maintenance organizations. They aggregated 3-digit codes of the *ICD-9-CM* to create what they termed a manageable number of clinically meaningful subgroups for empirical analyses. In 1996, Ellis and colleagues^24^ showed that hierarchical coexisting condition models achieved greater explanatory power than the diagnostic cost group models. They identified 143 inpatient groupings and 432 groupings from hospital and physician diagnoses. In 2000, Pope and colleagues^1^ produced an iteration on the diagnostic cost group HCC models for Medicare risk adjustment. In this iteration, the classification system underwent further refinement, using 189 groupings of diagnoses and instituting a hierarchy of codes. In 2015, CMS developed a model using these condition categories to support public reporting of risk-standardized Medicare payments at hospitals across the United States.^8,9,12^ Our findings suggest that there are opportunities for improvements in the predictive performance of these models with relatively simple changes in the candidate variables.

The improvements in the model performance were meaningful. For example, the upper range of prediction for patients with AMI increased from $55 387 to $113 446. The estimate on the lower end declined from $10 993 to $8204. This change was consistent in all the models. The difference revealed that the new models were better able to identify people at the ends of the payment spectrum. We also showed that a substantial number of patients had different predicted payment estimates with the new model, including approximately 5% who moved from being predicted to be in the lowest quintile to the highest quintile and another 5% who moved from being predicted to be in the highest quintile to the lowest quintile of payment.

A key consideration of all these models was the prevention of gaming. With the use of individual codes, it may have been easier for institutions to focus on attributing higher-risk codes to the patient population than might be possible with the grouped codes. However, it was also possible to monitor for changes in individual codes and update models on a routine basis. As the importance of diagnoses change, the models can be modified.

There are implications to these findings. In an exploratory analysis, we showed that the better models changed the assessment of some hospitals. Many hospitals that the models classified as better or worse than the national mean payment changed categories, even as the overall percentage that the models identified as significantly different from the mean changed only slightly. The presumption was that the better performing model was more appropriately identifying outliers by better accounting for the case mix of the hospitals. This finding may represent an opportunity to improve the models for the future. Some additional steps are necessary, however, including the testing of this approach with the *International Statistical Classification of Diseases, Tenth Revision, Clinical Modification (ICD-10-CM)* codes. In addition, this approach is relevant to models being used now to calculate Medicare Advantage payments. With a rapidly increasing number of beneficiaries enrolled in Medicare Advantage, the importance of risk models to determine payment is growing.^25^ Better models could also have relevance to planning at the health system level as the focus on population health increases and the need to identify people with the highest risk of incurring costs increases.

### Limitations

This study has several limitations. The study used the last cohort with *ICD-9-CM* codes to have enough patients for the development and testing of the approach. As more data with *ICD-10-CM* codes accumulate, there will be a need to develop and test this approach with the larger number of codes that are available. Nevertheless, these data experiments expose the possibilities of changing our approach to claims-based risk adjustment. The study also focused on the candidate variables and did not explore variations of the analytic methods. Although more sophisticated machine learning approaches could have been used for variable selection, we chose to use LASSO because of its ease in selecting a specified number of codes as required by our hospital-level algorithm and to avoid possible issues that multicollinearity can cause in a hierarchical GLM.^19^ With LASSO, some relevant codes might be dropped because of correlation and there may be diminished predictive power as a result. Nevertheless, we were consistent in our approach in all the new models; thus, it should not have biased our assessment. Also, the variables used in these models differed from those used in many clinically based risk-stratification approaches. This study was specifically about the use of claims-based candidate variables. Currently, we lack more detailed clinical information on a national basis, and thus only these data are routinely available to CMS. In testing different approaches, we restricted ourselves to the same variables that are used in the current CMS models. The importance of the magnitude of improvement is challenging to put in perspective. We showed that the new model is better at predicting the tails of the distribution. These improvements, given that there is no marginal cost to producing the estimates, seem worthwhile.

## Conclusions

In conclusion, we introduced a new way to approach modeling payment for Medicare beneficiaries. We tested various approaches to the use of candidate variables while holding constant the source data, the diagnoses, and the analytic methods. We showed that by using individual codes, leveraging the POA designations, and separating index from historical codes, we could improve the performance of the models. The findings open the possibility of improving research, performance assessment, and payment determinations by improving characterizations of case mix as well as improving population health by being better able to identify individuals at high risk of incurring high costs.

## Notes

Supplement. **eAppendix.** POA-exempt *ICD* Code Methodology **eTable 1.** List of the Top 50 *ICD-9-CM* Codes or Other Variables Selected by the Individual-codes Model for Acute Myocardial Infarction, as Ranked by Variable Importance (Computed by the Caret R Package) **eTable 2.** List of the Top 50 *ICD-9-CM* Codes or other Variables Selected by the Individual-codes Model for Heart Failure, as Ranked by Variable Importance (Computed by the Caret R Package) **eTable 3.** List of the Top 50 *ICD-9-CM* Codes or Other Variables Selected by the Individual-codes Model for Pneumonia, as Ranked by Variable Importance (Computed by the Caret R Package) **eTable 4.** Shift Table for Acute Myocardial Infarction Comparing the Predicted Payment of the Current CMS Model With the Individual-codes Model Across Quintiles. **eTable 5.** Shift Table for Heart Failure Comparing the Predicted Payment of the Current CMS Model With the Individual-codes Model Across Quintiles. **eTable 6.** Shift Table for Pneumonia Comparing the Predicted Payment of the Current CMS Model With the Individual-codes Model Across Quintiles. **eTable 7.** Distribution of Hospital-level Performance Categories Comparing Publicly Reported CMS Models to Individual-codes Models Incorporating Proposed Patient-level Model Changes for Acute Myocardial Infarction, Heart Failure, and Pneumonia 30-Day Payment Measures, Among Hospitals With at Least 25 Cases **eTable 8.** CMS Publicly Reported Performance “Categories” for CMS Models Compared to Individual-codes Models for Acute Myocardial Infarction, Heart Failure, and Pneumonia 30-Day Payment Measures, Among Hospitals With at Least 25 Cases
