# Supplementary material for: Development and Testing of Improved Models to Predict Payment Using Centers for Medicare & Medicaid Services Claims Data
Source: JAMA Netw Open. 2019 Aug 14;2(8):e198406. doi: 10.1001/jamanetworkopen.2019.8406 (PMC6694388; doi:10.1001/jamanetworkopen.2019.8406)
Supplement: Supplement. — eAppendix. POA-exempt ICD Code Methodology eTable 1. List of the Top 50 ICD-9-CM Codes or Other Variables Selected by the Individual-codes Model for Acute Myocardial Infarction, as Ranked by Variable Importance (Computed by the Caret R Package) eTable 2. List of the Top 50 ICD-9-CM Codes or other Variables Selected by the Individual-codes Model for Heart Failure, as Ranked by Variable Importance (Computed by the Caret R Package) eTable 3. List of the Top 50 ICD-9-CM Codes or Other Variables Selected by the Individual-codes Model for Pneumonia, as Ranked by Variable Importance (Computed by the Caret R Package) eTable 4. Shift Table for Acute Myocardial Infarction Comparing the Predicted Payment of the Current CMS Model With the Individual-codes Model Across Quintiles. eTable 5. Shift Table for Heart Failure Comparing the Predicted Payment of the Current CMS Model With the Individual-codes Model Across Quintiles. eTable 6. Shift Table for Pneumonia Comparing the Predicted Payment of the Current CMS Model With the Individual-codes Model Across Quintiles. eTable 7. Distribution of Hospital-level Performance Categories Comparing Publicly Reported CMS Models to Individual-codes Models Incorporating Proposed Patient-level Model Changes for Acute Myocardial Infarction, Heart Failure, and Pneumonia 30-Day Payment Measures, Among Hospitals With at Least 25 Cases eTable 8. CMS Publicly Reported Performance “Categories” for CMS Models Compared to Individual-codes Models for Acute Myocardial Infarction, Heart Failure, and Pneumonia 30-Day Payment Measures, Among Hospitals With at Least 25 Cases [file jamanetwopen-2-e198406-s001.pdf]

Supplementary Online Content

Krumholz HM, Warner F, Coppi A, et al. Development and testing of improved models to predict payment using Centers for Medicare & Medicaid Services claims data. *JAMA Netw Open*. 2019;2(8):e198406. doi:10.1001/jamanetworkopen.2019.8406

eAppendix. POA-exempt *ICD* Code Methodology

eTable 1. List of the Top 50 *ICD-9-CM* Codes or Other Variables Selected by the Individual-codes Model for Acute Myocardial Infarction, as Ranked by Variable Importance (Computed by the Caret R Package)

eTable 2. List of the Top 50 *ICD-9-CM* Codes or other Variables Selected by the Individual-codes Model for Heart Failure, as Ranked by Variable Importance (Computed by the Caret R Package)

eTable 3. List of the Top 50 *ICD-9-CM* Codes or Other Variables Selected by the Individual-codes Model for Pneumonia, as Ranked by Variable Importance (Computed by the Caret R Package)

eTable 4. Shift Table for Acute Myocardial Infarction Comparing the Predicted Payment of the Current CMS Model With the Individual-codes Model Across Quintiles

eTable 5. Shift Table for Heart Failure Comparing the Predicted Payment of the Current CMS Model With the Individual-codes Model Across Quintiles

eTable 6. Shift Table for Pneumonia Comparing the Predicted Payment of the Current CMS Model With the Individual-codes Model Across Quintiles

eTable 7. Distribution of Hospital-level Performance Categories Comparing Publicly Reported CMS Models to Individual-codes Models Incorporating Proposed Patient-level Model Changes for Acute Myocardial Infarction, Heart Failure, and Pneumonia 30-Day Payment Measures, Among Hospitals With at Least 25 Cases

eTable 8. CMS Publicly Reported Performance “Categories” for CMS Models Compared to Individual-codes Models for Acute Myocardial Infarction, Heart Failure, and Pneumonia 30-Day Payment Measures, Among Hospitals With at Least 25 Cases

This supplementary material has been provided by the authors to give readers additional information about their work.

© 2019 Krumholz HM et al. *JAMA Network Open*

eAppendix. POA-exempt *ICD* Code Methodology

As part of a currently unpublished separate project, we convened a technical working group (TWG) of clinical experts who provided clinical rationale for categorizing the POA-exempt codes as “always POA” or “don’t count as POA” based on their clinical expertise, the rationale from the official ICD-9-CM and ICD-10-CM coding guidelines, and our recommendations. The majority of the ICD-9-CM and ICD-10-CM codes included in the POA-exempt list were subsequent, sequela, or congenital codes. Based on ICD-9-CM and ICD-10-CM coding guidelines, we coded these types of codes as “always POA” because, by definition, subsequent and sequela codes should not be used for conditions acquired during a hospitalization in which the patient is receiving active treatment for that condition. Additional groups of codes were further reviewed by clinical experts to determine whether they should be counted as “always POA” or not. We excluded POA-exempt codes from the “always POA” list based on any one of four criteria: 1) they were not relevant to the Medicare population, such as codes related to pregnancy, childbirth, and children’s health statuses; 2) they could potentially be coded as a complication of care during an index admission or POA, such as exposure to toxic substances or medication overdoses; 3) they provided no relevant information about a patient’s health status or reason for admission, such as family history codes or encounter codes, which indicate that a patient has an encounter for a procedure but does not specify that the procedure was performed; or 4) they were not mandatory for reporting. The latter exclusion pertained specifically to S00-T88 Injury, poisoning, and certain other consequences of external causes and V00-Y99 External Causes of Morbidity, which are claims collected for the purposes of injury research.

The lists of ICD-9-CM POA-exempt codes resulting from the above were used as the basis for determining which of a patient’s diagnosis codes neither flagged POA=Y nor POA=N would be allowable data for the individual codes model fit.

The project was conducted by the Yale New Haven Health Services Corporation–Center for Outcomes Research and Evaluation (CORE). The research was led by the following individuals affiliated with CORE:

Elizabeth W. Triche, PhD

Shengfan Zhou, MS

Danielle Purvis, MPH

Grace Glennon, MS

Kofi Dwamena, BS

© 2019 Krumholz HM et al. *JAMA Network Open*

eTable 1. List of the Top 50 *ICD-9-CM* Codes or Other Variables Selected by the Individual-codes Model for Acute Myocardial Infarction, as Ranked by Variable Importance (Computed by the Caret R Package)

ICD-9-CM / Variable Name	Description	Frequency
V4986	Do not resuscitate status	9.56
41401	Coronary atherosclerosis of native coronary artery	67.15
age_admit_3	Discretized age	n/a
78551	Cardiogenic shock	4.41
42983	Takotsubo syndrome	1.23
42821	Acute systolic heart failure	4.93
4280	Congestive heart failure, unspecified	36.76
42823	Acute on chronic systolic heart failure	6.54
4148	Other specified forms of chronic ischemic heart disease	14.24
42741	Ventricular fibrillation	1.51
5856	End stage renal disease	4.07
51881	Acute respiratory failure	7.96
4240	Mitral valve disorders	8.03
4241	Aortic valve disorders	7.30
V4582	Percutaneous transluminal coronary angioplasty status	17.09
43310	Occlusion and stenosis of carotid artery without mention of cerebral infarction	3.35
486	Pneumonia, organism unspecified	6.39
42843	Acute on chronic combined systolic and diastolic heart failure	2.58
HxP_25000	Diabetes mellitus without mention of complication, type II or unspecified type, not stated as uncontrolled	37.34
V4502	Automatic implantable cardiac defibrillator in situ	2.56
4260	Atrioventricular block, complete	1.12
2639	Unspecified protein-calorie malnutrition	1.26
HxP_V4581	Aortocoronary bypass status	11.91
42833	Acute on chronic diastolic heart failure	3.66
4271	Paroxysmal ventricular tachycardia	3.32
42841	Acute combined systolic and diastolic heart failure	0.90
43491	Cerebral artery occlusion, unspecified with cerebral infarction	0.50
5119	Unspecified pleural effusion	1.60
41402	Coronary atherosclerosis of autologous vein bypass graft	5.17
42831	Acute diastolic heart failure	1.72
5845	Acute kidney failure with lesion of tubular necrosis	1.28
HxP_V7612	Other screening mammogram	8.89
4144	Coronary atherosclerosis due to calcified coronary lesion	1.21
V4501	Cardiac pacemaker in situ	5.34
2761	Hyposmolality and/or hyponatremia	5.64
262	Other severe protein-calorie malnutrition	0.59

© 2019 Krumholz HM et al. *JAMA Network Open*

eTable 1. (Continued)

ICD-9-CM / Variable Name	Description	Frequency
27801	Morbid obesity	3.48
72888	Rhabdomyolysis	1.12
2851	Acute posthemorrhagic anemia	1.44
42781	Sinoatrial node dysfunction	1.47
7993	Debility, unspecified	2.27
V5866	Long-term (current) use of aspirin	19.49
2875	Thrombocytopenia, unspecified	3.49
42789	Other specified cardiac dysrhythmias	6.86
HxP_78650	Chest pain, unspecified	32.42
HxP_41401	Coronary atherosclerosis of native coronary artery	34.55
4400	Atherosclerosis of aorta	0.83
412	Old myocardial infarction	13.74
4142	Chronic total occlusion of coronary artery	10.21
5070	Pneumonitis due to inhalation of food or vomitus	1.05

A prepended "Hx_" on the ICD-9-CM code label indicates that it occurred during the 12-prior claim history; otherwise, the code is from the index admission.

ICD-9-CM, International Classification of Diseases, Ninth Revision, Clinical Modification

© 2019 Krumholz HM et al. *JAMA Network Open*

eTable 2. List of the Top 50 *ICD-9-CM* Codes or Other Variables Selected by the Individual-codes Model for Heart Failure, as Ranked by Variable Importance (Computed by the caret R Package)

ICD-9-CM / Variable Name	Description	Frequency
486	Pneumonia, organism unspecified	13.84
51881	Acute respiratory failure	12.72
51884	Acute and chronic respiratory failure	6.41
41071	Subendocardial infarction, initial episode of care	2.25
5856	End stage renal disease	5.10
V4502	Automatic implantable cardiac defibrillator in situ	8.52
40491	Hypertensive heart and chronic kidney disease, unspecified, with heart failure and with chronic kidney disease stage I through stage IV, or unspecified	5.28
4241	Aortic valve disorders	9.95
4148	Other specified forms of chronic ischemic heart disease	14.01
4254	Other primary cardiomyopathies	14.91
5119	Unspecified pleural effusion	6.67
4271	Paroxysmal ventricular tachycardia	2.67
4142	Chronic total occlusion of coronary artery	0.85
4263	Other left bundle branch block	4.23
5845	Acute kidney failure with lesion of tubular necrosis	0.85
7993	Debility, unspecified	4.28
2761	Hyposmolality and/or hyponatremia	9.03
41401	Coronary atherosclerosis of native coronary artery	33.39
V4986	Do not resuscitate status	12.74
5849	Acute kidney failure, unspecified	20.62
78551	Cardiogenic shock	0.58
42781	Sinoatrial node dysfunction	2.24
5990	Urinary tract infection, site not specified	9.82
age_admit_3	Discretized age	n/a
V1581	Personal history of noncompliance with medical treatment, presenting hazards to health	6.12
42822	Chronic systolic heart failure	0.83
6826	Cellulitis and abscess of leg, except foot	2.86
V4501	Cardiac pacemaker in situ	13.73
262	Other severe protein-calorie malnutrition	0.90
4111	Intermediate coronary syndrome	1.01
3962	Mitral valve insufficiency and aortic valve stenosis	2.31
34831	Metabolic encephalopathy	1.11
2639	Unspecified protein-calorie malnutrition	1.87
42732	Atrial flutter	3.82
HxP_1101	Dermatophytosis of nail	22.92
70703	Pressure ulcer, low back	1.61
V1588	Personal history of fall	2.37

© 2019 Krumholz HM et al. *JAMA Network Open*

eTable 2. (Continued)

ICD-9-CM / Variable Name	Description	Frequency
5070	Pneumonitis due to inhalation of food or vomitus	0.70
34830	Encephalopathy, unspecified	1.04
27803	Obesity hypoventilation syndrome	1.49
7812	Abnormality of gait	1.37
4019	Unspecified essential hypertension	34.72
2851	Acute posthemorrhagic anemia	0.96
4168	Other chronic pulmonary heart diseases	21.58
HxP_4241	Aortic valve disorders	17.92
49121	Obstructive chronic bronchitis with (acute) exacerbation	9.66
78079	Other malaise and fatigue	2.87
HxP_V4986	Do not resuscitate status	9.07
V5866	Long-term (current) use of aspirin	16.74
V4582	Percutaneous transluminal coronary angioplasty status	13.13

A prepended “Hx_” on the ICD-9-CM code label indicates that it occurred during the 12-prior claim history; otherwise, the code is from the index admission.

ICD-9-CM, International Classification of Diseases, Ninth Revision, Clinical Modification

© 2019 Krumholz HM et al. *JAMA Network Open*

eTable 3. List of the Top 50 *ICD-9-CM* Codes or Other Variables Selected by the Individual-codes Model for Pneumonia, as Ranked by Variable Importance (Computed by the Caret R Package)

ICD-9-CM / Variable Name	Description	Frequency
51881	Acute respiratory failure	14.25
51884	Acute and chronic respiratory failure	7.49
5119	Unspecified pleural effusion	6.39
5070	Pneumonitis due to inhalation of food or vomitus	17.85
48242	Methicillin resistant pneumonia due to Staphylococcus aureus	1.60
262	Other severe protein-calorie malnutrition	2.47
34831	Metabolic encephalopathy	3.71
7993	Debility, unspecified	5.18
4280	Congestive heart failure, unspecified	29.96
2639	Unspecified protein-calorie malnutrition	3.86
34830	Encephalopathy, unspecified	2.98
99591	Sepsis	22.43
5845	Acute kidney failure with lesion of tubular necrosis	0.95
5990	Urinary tract infection, site not specified	14.81
41071	Subendocardial infarction, initial episode of care	1.76
4821	Pneumonia due to Pseudomonas	1.35
HxP_V4986	Do not resuscitate status	9.01
34982	Toxic encephalopathy	1.78
2859	Anemia, unspecified	15.63
5849	Acute kidney failure, unspecified	16.75
42833	Acute on chronic diastolic heart failure	3.53
5856	End stage renal disease	3.71
2851	Acute posthemorrhagic anemia	1.06
41519	Other pulmonary embolism and infarction	0.61
2761	Hyposmolality and/or hyponatremia	11.58
42823	Acute on chronic systolic heart failure	2.05
0389	Unspecified septicemia	20.81
HxP_V7612	Other screening mammogram	8.28
48241	Methicillin susceptible pneumonia due to Staphylococcus aureus	0.51
72888	Rhabdomyolysis	0.83
29420	Dementia, unspecified, without behavioral disturbance	14.91
5180	Pulmonary collapse	3.49
48283	Pneumonia due to other gram-negative bacteria	2.68
0380	Streptococcal septicemia	0.56
42731	Atrial fibrillation	28.79
28529	Anemia of other chronic disease	5.15
78720	Dysphagia, unspecified	8.32
HxP_V5811	Encounter for antineoplastic chemotherapy	3.00
HxP_1101	Dermatophytosis of nail	23.87
V440	Tracheostomy status	0.73

© 2019 Krumholz HM et al. *JAMA Network Open*

eTable 3. (Continued)

ICD-9-CM / Variable Name	Description	Frequency
HxP_7812	Abnormality of gait	12.34
261	Nutritional marasmus	0.53
2762	Acidosis	4.82
42831	Acute diastolic heart failure	1.07
HxP_7866	Swelling, mass, or lump in chest	4.18
HxP_72887	Muscle weakness (generalized)	13.34
34839	Other encephalopathy	0.61
V5866	Long-term (current) use of aspirin	13.06
4168	Other chronic pulmonary heart diseases	6.58
42732	Atrial flutter	1.72

A prepended “Hx_” on the ICD-9-CM code label indicates that it occurred during the 12-prior claim history; otherwise, the code is from the index admission.

ICD-9-CM, International Classification of Diseases, Ninth Revision, Clinical Modification

© 2019 Krumholz HM et al. *JAMA Network Open*

eTable 4. Shift Table for Acute Myocardial Infarction Comparing the Predicted Payment of the Current CMS Model With the Individual-codes Model Across Quintiles. (Each cell contains the number of index admissions in the intersection of the 2 models' predicted payment categories [row vs col], and below it, within square brackets, is the observed mean Winsorized payment for that subset of index admissions.)

**Acute myocardial infarction** No. of patients [mean observed payment]		**CMS model predicted payment (quintiles)**					**Row totals**
		**1st**	**2nd**	**3rd**	**4th**	**5th**	
**Individual-codes model predicted payment (quintiles)**	**1st**	31,893 [$15,492]	16,166 [$16,248]	9827 [$17,002]	7300 [$17,645]	3458 [$19,027]	68,644 [$16,293]
	**2nd**	16,872 [$18,074]	20,034 [$18,233]	14,064 [$18,718]	11,429 [$20,540]	6269 [$23,460]	68,668 [$19,154]
	**3rd**	8882 [$19,660]	14,056 [$20,057]	19,357 [$19,581]	15,405 [$21,648]	10,868 [$26,757]	68,568 [$21,291]
	**4th**	7169 [$21,816]	10,949 [$23,289]	14,747 [$22,961]	18,238 [$24,603]	17,513 [$30,181]	68,616 [$25,173]
	**5th**	3807 [$25,798]	7422 [$28,716]	10,632 [$30,075]	16,244 [$31,897]	30,515 [$37,554]	68,620 [$33,448]
**Column totals**		68,623 [$17,899]	68,627 [$20,079]	68,627 [$21,387]	68,616 [$24,249]	68,623 [$31,741]	343,116 [$23,071]

CMS, Centers for Medicare & Medicaid Services

© 2019 Krumholz HM et al. *JAMA Network Open*

eTable 5. Shift Table for Heart Failure Comparing the Predicted Payment of the Current CMS Model With the Individual-codes Model Across Quintiles (Each cell contains the number of index admissions in the intersection of the 2 models' predicted payment categories [row vs col], and below it, within square brackets, is the observed mean Winsorized payment for that subset of index admissions.)

**Heart failure** No. of patients [mean observed payment]		**CMS model predicted payment (quintiles)**					**Row totals**
		**1st**	**2nd**	**3rd**	**4th**	**5th**	
**Individual-codes model predicted payment (quintiles)**	**1st**	56,141 [$10,976]	35,506 [$11,409]	24,015 [$11,792]	14,433 [$12,230]	5314 [$13,054]	135,409 [$11,450]
	**2nd**	35,491 [$13,577]	34,540 [$13,676]	30,088 [$13,851]	23,440 [$14,105]	11,850 [$14,895]	135,409 [$13,870]
	**3rd**	23,697 [$15,589]	30,020 [$15,666]	31,623 [$15,959]	30,117 [$16,042]	19,951 [$16,498]	135,408 [$15,927]
	**4th**	14,282 [$17,957]	22,863 [$17,871]	29,256 [$18,008]	34,898 [$18,055]	34,110 [$18,381]	135,409 [$18,085]
	**5th**	5913 [$21,365]	12,399 [$21,517]	20,394 [$21,234]	32,519 [$21,487]	64,184 [$22,436]	135,409 [$21,896]
**Column totals**		135,524 [$13,653]	135,328 [$14,950]	135,376 [$15,989]	135,407 [$17,127]	135,409 [$19,512]	677,044 [$16,246]

CMS, Centers for Medicare & Medicaid Services

eTable 6. Shift Table for Pneumonia Comparing the Predicted Payment of the Current CMS Model With the Individual-codes Model Across Quintiles (Each cell contains the number of index admissions in the intersection of the 2 models' predicted payment categories [row vs col], and below it, within square brackets, is the observed mean Winsorized payment for that subset of index admissions.)

**Pneumonia** No. of patients [mean observed payment]		CMS model predicted payment (quintiles)					**Row totals**
		**1st**	**2nd**	**3rd**	**4th**	**5th**	
**Individual-codes model predicted payment (quintiles)**	**1st**	95,960 [$9401]	50,060 [$10,855]	25,493 [$11,800]	10,417 [$12,675]	2648 [$13,444]	184,578 [$10,369]
	**2nd**	47,708 [$13,246]	52,601 [$14,065]	43,595 [$14,409]	29,213 [$14,775]	11,461 [$15,209]	184,578 [$14,118]
	**3rd**	24,409 [$16,058]	41,878 [$16,555]	48,325 [$17,013]	44,482 [$17,190]	25,483 [$17,282]	184,577 [$16,863]
	**4th**	12,083 [$18,893]	27,552 [$19,183]	41,810 [$19,436]	53,798 [$19,734]	49,335 [$20,076]	184,578 [$19,621]
	**5th**	4418 [$23,616]	12,487 [$23,006]	25,354 [$23,068]	46,668 [$23,622]	95,651 [$25,393]	184,578 [$24,422]
**Column totals**		184,578 [$12,237]	184,578 [$15,128]	184,577 [$17,059]	184,578 [$18,921]	184,578 [$22,048]	922,889 [$17,079]

CMS, Centers for Medicare & Medicaid Services

© 2019 Krumholz HM et al. *JAMA Network Open*

eTable 7. Distribution of Hospital-level Performance Categories Comparing Publicly Reported CMS Models to Individual-codes Models Incorporating Proposed Patient-level Model Changes for Acute Myocardial Infarction, Heart Failure, and Pneumonia 30-Day Payment Measures, Among Hospitals With at Least 25 Cases

	Lower than national payment, n (%)	No different than national payment, n (%)	Higher than national payment, n (%)
**Acute myocardial infarction payment (N=2181)**			
CMS model	161 (7.4)	1861 (85.3)	159 (7.3)
Individual-codes model	227 (10.4)	1823 (83.6)	131 (6.0)
**Heart failure payment (N=3265)**			
CMS model	287 (8.8)	2538 (77.7)	440 (13.5)
Individual-codes model	320 (9.8)	2615 (80.1)	330 (10.1)
**Pneumonia payment (N=3831)**			
CMS model	713 (18.6)	2435 (63.6)	683 (17.8)
Individual-codes model	807 (21.1)	2544 (66.4)	480 (12.5)

CMS, Centers for Medicare & Medicaid Services

© 2019 Krumholz HM et al. *JAMA Network Open*

eTable 8. CMS Publicly Reported Performance "Categories" for CMS Models Compared to Individual-codes Models for Acute Myocardial Infarction, Heart Failure, and Pneumonia 30-Day Payment Measures, Among Hospitals With at Least 25 Cases

	**Individual code buckets**			
**CMS model buckets**	Lower than national payment	No different than national payment	Higher than national payment	Total
**Acute myocardial infarction performance bucket shift**				
Lower than the national payment	137	24	0	161
No different than the national payment	90	1726	45	1861
Higher than the national payment	0	73	86	159
Total	227	1823	131	2181
**Heart failure performance bucket shift**				
Lower than the national payment	190	97	0	287
No different than the national payment	130	2355	53	2538
Higher than the national payment	0	163	277	440
Total	320	2615	330	3265
**Pneumonia performance bucket shift**				
Lower than the national payment	428	285	0	713
No different than the national payment	365	1924	146	2435
Higher than the national payment	14	335	334	683
Total	807	2544	480	3831

CMS, Centers for Medicare & Medicaid Services

© 2019 Krumholz HM et al. *JAMA Network Open*
